# Low potassium intake and its association with blood pressure among adults in Malaysia: findings from the MyCoSS (Malaysian Community Salt Survey)

**DOI:** 10.1186/s41043-021-00238-x

**Published:** 2021-05-31

**Authors:** Lalitha Palaniveloo, Rashidah Ambak, Fatimah Othman, Nor Azian Mohd Zaki, Azli Baharudin, Nur Shahida Abdul Aziz, Ruhaya Salleh

**Affiliations:** 1 Institute for Public Health, National Institutes of Health, Ministry of Health Malaysia, Selangor, Malaysia; 2 Department of Dietetic and Food Service, Sarawak General Hospital, Ministry of Health Malaysia, Kuching, Sarawak Malaysia

**Keywords:** Potassium, Adults, Blood pressure, Malaysia

## Abstract

**Background:**

High blood pressure or hypertension is well recognized as an important modifiable risk factor for cardiovascular diseases. Several studies had indicated potassium intake has a blood pressure lowering effect. This study aimed to estimate potassium intake via 24-h urinary potassium excretion and to determine the association between potassium intake and blood pressure among adults in Malaysia.

**Methods:**

Data for 424 respondents in this study were drawn from MyCoSS, a nationwide cross- sectional study conducted among Malaysians who were 18 years and above. Respondents were recruited using stratified cluster sampling, covering urban and rural areas in each state in Malaysia. Data collection was undertaken from October 2017 until March 2018. A single urine sample was collected over 24 h for quantification of potassium excreted. Information on socio-demography and medical history of the respondents were collected by interviewer-administered questionnaires. Anthropometric measurements were measured using validated equipment. BMI was estimated using measured body weight and height. Digital blood pressure monitor (Omron HBP-1300) was used to measure blood pressure. Descriptive statistics, analysis of variance (ANOVA), and multivariable linear regression were used to analyze the data in SPSS Version 21.

**Results:**

Mean 24-h urinary potassium excretion for the 424 respondents was 37 mmol (95% CI 36, 38). Gender and ethnicity showed statistically significant associations with 24-h urinary potassium excretion. However, potassium excretion was not significantly associated with blood pressure in this study.

**Conclusion:**

Potassium intake is very low among the adults in Malaysia. Therefore, further education and promotional campaigns regarding daily consumption of potassium-rich diet and its benefits to health need to be tailored for the Malaysian adult population.

## Background

Nearly 1 billion people in the world are affected by high blood pressure or hypertension and it is recognized as an important modifiable risk factor for cardiovascular diseases. High blood pressure affects one out of three adults in South-East Asian countries, a condition identified to be the leading cause for an estimation of 1.5 million deaths in those countries [1]. In 2015, the prevalence of hypertension was 30.3% among adults who were 18 years old and above in Malaysia [2]. World Health Organization (WHO) in their report Global Atlas on Cardiovascular Disease Prevention and Control (2011) had stated that WHO African region recorded the highest prevalence of high blood pressure among their adults with 46% while the lowest was in WHO Americas region with 35% [3].

Various factors such as unhealthy dietary practices, smoking, obesity, and physical inactivity affect blood pressure [4]. With regard to potassium, this mineral is important for maintaining total body fluid, nerve impulses transmission, heart beat (rhythm) maintenance, and muscle contractions [5, 6]. High potassium intake also has been associated with blood pressure lowering effect among those with elevated blood pressure, reducing cardiovascular-related mortality, preventing or slowing down the advancement of renal disease and lowering urinary calcium excretion, thereby, reducing the risk of osteoporosis [7, 8]. In addition, increased potassium intake alleviates the negative effects of high sodium consumption on blood pressure [9, 10]. A possible mechanism for potassium’s effect on blood pressure is explained by its role in increasing the amount of sodium excreted via urine, which in return, reduces the availability of sodium in the body. This will lead to relaxation of vascular smooth muscles, thus lowering the blood pressure [10]. These findings on the protective effects of potassium intake led to the WHO recommendation of a daily potassium intake of 90 mmol (3510 mg) for adults [11]. However, the 2017 Malaysian Recommended Nutrient Intake (RNI) for potassium for adults > 18 years old is slightly higher at 120 mmol (4700 mg) per day [12]. This is in line with the Institute of Medicine’s (IOM) recommendation on adequate daily potassium intake for adults [13].

Estimating potassium intake via potassium excreted in 24-h urine samples is a very reliable method in comparison with dietary assessment. Potassium is excreted from the body through urine (90%), stool (10%) and sweat (very little) [14]. Conversely, estimation of potassium intake through dietary assessment is prone for recall bias from the respondents which can lead to under or over estimation of the nutrient intake [15].

Currently, Malaysia has very limited data regarding potassium intake. The only data available is for adolescents which was determined through dietary intake [16]. Moreover, studies conducted worldwide showed mixed views on association between potassium intake and blood pressure [9, 10, 17–20]. In view of this and the steady increase in prevalence of hypertension among the adults, there is an urgent need for baseline data on potassium intake in Malaysia. Therefore, this study aimed to estimate potassium intake via 24-h urinary potassium excretion among adults in Malaysia. A secondary aim was to assess the relationship between potassium intake and blood pressure.

## Methods

Data for this study were drawn from MyCoSS, which was a nationwide cross-sectional study targeting Malaysian adults who were 18 years and above who lived in non-institutional living quarters (LQ). The sample size was calculated using a formula for estimating mean, using the mean 24-h urinary potassium excretion value obtained from MySalt Study (2012) of 35.57 mmol/L (17.12) [Institute for Public Health. Determination of Dietary Sodium Intake among the Ministry of Health Staff (MySalt 2012). Ministry of Health, Malaysia. 2012 (Unpublished)], precision between 4 and 12, estimated design effect of 1.5 and with consideration of 60% non-response rate. The respondents were recruited using stratified cluster sampling method, covering both urban and rural areas in each state in Malaysia. Data collection was undertaken from October 2017 until March 2018. Inclusion criteria was Malaysian adults who were 18 years old and above. Those who were fasting during data collection, on diuretic therapy (< 4 weeks), in any situation which will results in difficulties for 24-h urine collection, pregnant, diagnosed with kidney disease, heart failure, or liver disease were excluded from this study.

### 24-h urine collection

Each participant was given detailed oral and printed instructions on collection of the 24-h urine accurately, together with a 5-L screw-capped plastic collection bottle, a urine collection jug, plastic carrier bags (for transporting the urine collection set), a safety pin (to be attached to undergarments, as a remainder for urine collection), and a poster. Respondents was advised to discard the first urine excreted in the morning and collected all urine throughout the 24-h duration. The last urine collected was the first urine excreted on the start of the second day. The respondents were required to record the timing of the first and last collection of 24-h urine. They were also required to inform the researcher on any missed urine collection. Urinary creatinine level and volume excreted were used as an indicator for completeness of urine collection. Samples were excluded if the urinary creatinine < 6 mmol/day for men or < 4 mmol/day for women, extreme outliers for urinary creatinine (i.e., > 3 SDs from the mean) or 24-h urine volume < 500 ml for both genders. Samples from respondents who reported of missing urine collection between the 24-h time period were also excluded from the study.

The collected urine samples were brought to the nearest branch of the laboratory assigned at each study site. In that laboratory, the urine volume was measured and 30 ml aliquot from each sample was preserved in a 4 °C refrigerator before being transported to the centralized laboratory in Shah Alam, Selangor, on the same day for analysis.

### Potassium measurement

Potassium excreted in the urine was measured using indirect ion-selective electrode method [21]. An electric potential voltage is developed across the potassium membranes between the reference and the measuring electrode in accordance with the Nernst equation. The tension developed is then compared with the previously determined calibrator voltages and converted to an ion concentration [21]. Creatinine was measured according to Kinetic Jaffe (alkaline picrate with Lloyd’s reagent) method. Both the analyses were conducted in the laboratory using Architect C machine (Abbott CI8200, IL, USA). The urinary samples were analyzed in duplicates and the coefficient of variation (CV) for potassium and creatinine were 12.2% and 5.5%, respectively.

### Anthropometric measurement

Digital weighing scale was used to measure body weight with an accuracy of 0.1 kg (TANITA HD-319, IL, USA). Stadiometer was used to measure height to the nearest 0.1 cm (SECA 213, CA, USA). An average of two measurements was recorded as the final reading for both parameters. Body mass index (BMI) was calculated by weight (kg) divided by the square of height (m^2^) and categorized according to WHO 2006 guidelines as follows: underweight (BMI < 18.5 kg/m^2^); normal (BMI 18.5–24.9 kg/m^2^); overweight (BMI 25.0–29.9 kg/m^2^); and obese (BMI ≥ 30 kg/m^2^).

### Blood pressure measurement

Digital blood pressure monitor was used to measure blood pressure (Omron HBP-1300, Kyoto, Japan). The sensitivity and specificity of the Omron HBP-1300 used were 86.2% and 98.0%, respectively. Blood pressure was measured in the morning during urine bottle collection from the participants’ house. The participants were to be seated and asked to rest for at least 5 min before the measurement starts. The cuff used was wrapped to a tightness that allows two fingers to be inserted in between the cuff and the arm. The arm was placed at the same height as the heart during measurement. Three measurements with an interval of one to 2 min between each measurement were taken. The average of the last two readings was recorded.

### Socio-demographic information

Interviewer administered questionnaire was used to gather information on medical history and socio-demographic factors: age, locality, race, education level, and household income.

### Statistical analysis

All variables were described using descriptive statistics. Analysis of variance (ANOVA) was applied to compare mean urinary potassium excretion between the characteristics of the respondents. Multivariable linear regression was performed to determine the relationship between urinary potassium excretion level and blood pressure adjusted for covariates known to be associated with blood pressure including gender, ethnic, monthly household income, and BMI. Statistical significance was set at *p* < 0.05. SPSS statistical software package version 21 was used for statistical analysis in this study (SPSS Inc., Chicago, IL, USA).

## Results

**Fig. 1:**
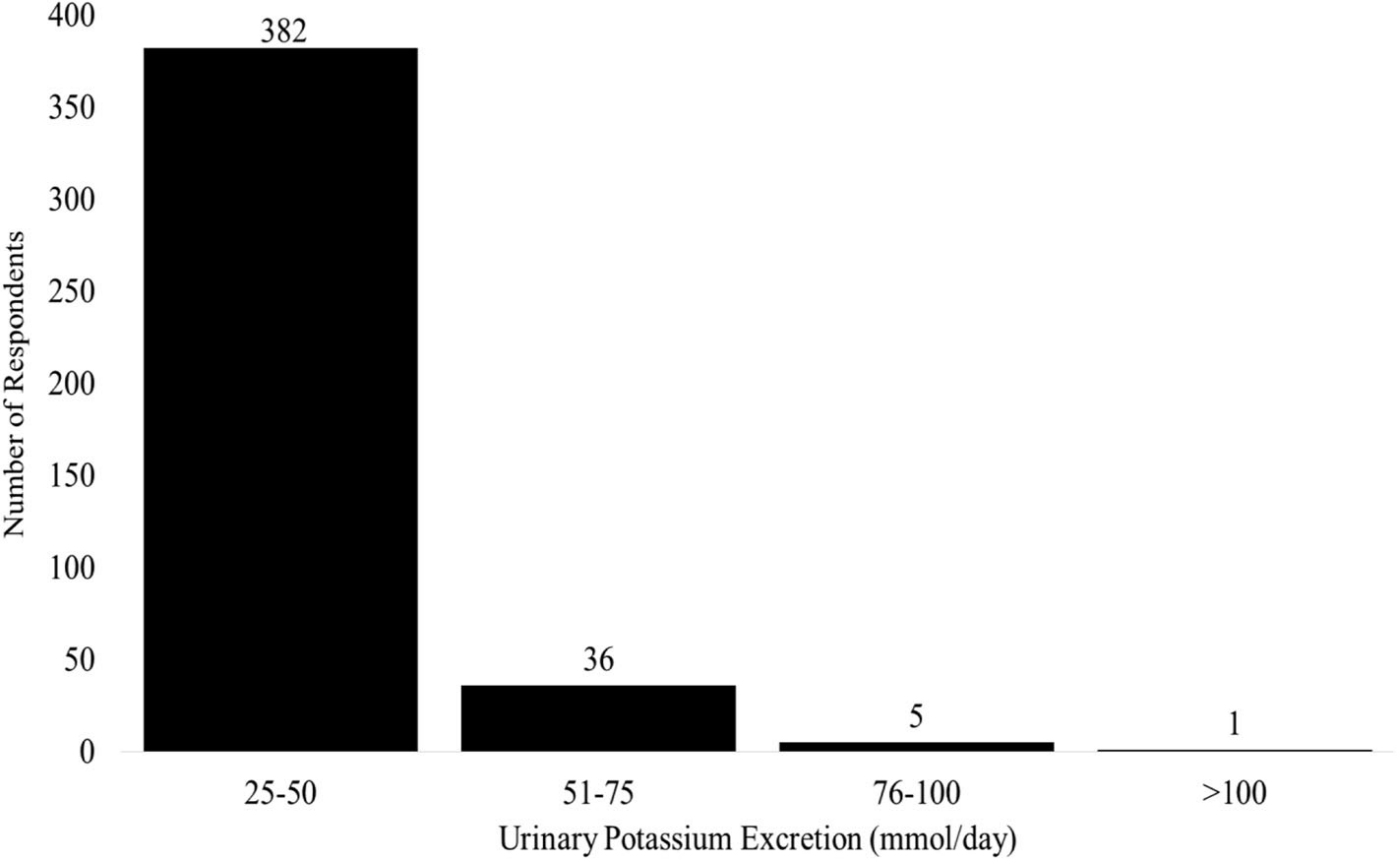
Distribution of 24-h potassium excretion of the respondents (*n* = 424)

A total of 424 respondents with urinary potassium excretion values were accepted for the analysis. The 24-h urinary potassium excretion of the respondents ranged between 25 mmol and 122 mmol with an overall mean of 37 mmol (95% CI 36, 38) (Fig. 1). Characteristics of the respondents are summarized in Table 1.

**Table 1:** Characteristics of the respondents by gender

Characteristic	Total (***N*** = 424)	Men (***N*** = 212)	Women (***N*** = 212)
	Mean (95% CI)/***n*** (%)		
**Age (years)**	49.1 (47.2, 50.9)	50.0 (47.7, 52.4)	47.7 (45.3, 50.2)
< 40	114 (27.6)	54 (27.8)	60 (27.3)
40–60	200 (46.5)	94 (41.4)	106 (53.2)
> 60	110 (25.9)	64 (30.8)	46 (19.5)
**Locality**			
Urban	181 (78.3)	92 (77.3)	89 (79.6)
Rural	243 (21.7)	120 (22.7)	123 (20.4)
**Ethnicity**			
Malay	267 (67.1)	135 (68.6)	132 (65.0)
Chinese	52 (13.1)	21 (10.7)	31 (16.4)
Indian	19 (7.9)	7 (6.1)	12 (10.4)
Others	86 (11.9)	49 (14.6)	37 (8.2)
**Education level**			
None	28 (3.2)	13 (3.0)	15 (3.4)
Primary	93 (19.3)	46 (17.2)	47 (22.2)
Secondary	198 (49.7)	98 (49.3)	100 (50.2)
Tertiary	105 (27.8)	55 (30.5)	50 (24.3)
**Monthly household income (RM)** ^a^	3888.5 (3174.5, 4602.6)	3993.6 (3206.8, 4780.3)	3749.2 (2889.3, 4609.0)
Low (< 3860)	305 (63.8)	153 (63.0)	152 (65.0)
Middle (3860–8319)	87 (26.2)	43 (26.9)	44 (25.4)
High (≥ 8320)	32 (9.9)	16 (10.1)	16 (9.6)
**Body mass index (BMI) (kg/m**^**2**^**)**	27.1 (26.5, 27.7)	27.0 (26.3, 27.7)	27.3 (26.3, 28.4)
Underweight (< 18.5)	10 (1.8)	5 (2.2)	5 (1.3)
Normal (18.5–24.9)	140 (33.9)	69 (30.4)	71 (38.5)
Overweight (25.0–29.9)	161 (39.7)	91 (42.3)	70 (36.3)
Obese (≥ 30.0)	113 (24.6)	47 (25.2)	66 (23.8)
**Blood pressure (mmHg)**			
Systolic	136.0 (133.5, 138.4)	136.7 (133.6, 139.9)	134.9 (131.1, 138.7)
Dystolic	81.8 (80.5, 83.0)	82.88 (81.0, 84.8)	80.3 (78.4, 82.1)
**24-h urine volume (ml/day)**	1654.2 (1526.6, 1781.7)	1704.5 (1548.2, 1860.7)	1587.5 (1398.8, 1776.2)
**24-h urinary creatinine (mg/day)**	1171.8 (1106.0, 1237.5)	1398.8 (1323.2, 1474.4)	870.9 (836.7, 905.2)

. ^a^ Economic Planning Unit, Prime Minister’s Department, 2014. 1 USD was equivalent to Malaysian Ringgit (MYR) 4.22 at the time of study

Table 2 shows the characteristics of the respondents by 24-h urinary potassium excretion. Men showed a significantly higher mean excretion of potassium (39 mmol; 95% CI 37, 40) compared with women (35 mmol; 95% CI 34, 38) (*p* = 0.003). Among the main ethnic groups, Malaysian Chinese had a significantly highest excretion of potassium with a mean of 42 mmol (95% CI 37, 47) (*p* = 0.019) while the Malays had the lowest mean excretion, 36 mmol (95% CI 35, 37). Comparison by gender and ethnicity showed statistically significant associations with 24-h urinary potassium excretion while age, locality, monthly household income, and BMI of the respondents were not significantly associated.

**Table 2:** Characteristics of the respondents by 24-h urinary potassium excretion

Characteristic	***n***	Potassium (mmol/24 h)	
		Mean (95% CI)	***p*** value
**Gender**			
Men	212	39 (37, 40)	0.003*
Women	212	35 (34, 38)	
**Age (years)**			
< 40	114	35 (33, 37)	0.145
40–60	200	38 (36, 40)	
> 60	110	37 (35, 40)	
**Locality**			
Urban	181	36 (35, 38)	0.349
Rural	243	37 (36, 39)	
**Ethnicity**			
Malay	267	36 (35, 37)	0.019*
Chinese	52	42 (37, 47)	
Indian	19	38 (33, 43)	
Others	86	37 (35, 39)	
**Monthly household income (RM)**			
Low (< 3860)	305	36 (35, 38)	0.287
Middle (3860–8319)	87	38 (35, 41)	
High (≥ 8320)	32	39 (34, 44)	
**Body mass index (BMI) (kg/m**^**2**^**)**			
Underweight (< 18.5)	10	34 (24, 44)	
Normal (18.5–24.9)	140	37 (35, 39)	
Overweight (25.0–29.9)	161	36 (34, 38)	0.634
Obese (≥ 30.0)	113	38 (36, 40)	

*p < 0.05

This study shows there was no significant association between urinary potassium excretion and blood pressure. After adjusting for gender, age, and household income, there was a reduction of 0.15 mmHg (95% CI − 0.309, 0.004; *p* = 0.09) in systolic blood pressure, and a 0.12-mmHg (95% CI − 0.219, − 0.016; *p* = 0.101) reduction in diastolic blood pressure per mmol increment in potassium excretion.

## Discussion

To date, this is the first study on 24-h urinary potassium excretion in Malaysia. Findings from this study serve as a proxy for daily potassium intake among the adults in Malaysia. Overall mean urinary potassium excretion of the respondents in this study was 37 mmol/day which is lower than the recommended daily potassium intake by WHO (90 mmol), RNI for Malaysia, and IOM (120 mmol) [11–13]. It is also considerably lower compared to other reports. The Prospective Urban and Rural Epidemiological Study (PURE) involving 101,945 adults from 17 countries around the world reported mean urinary potassium excretion of 54 mmol/day [22]. Likewise, National Health and Nutrition Examination Survey (NHANES 2014) reported mean urinary potassium excretion of 55 mmol/day among adults aged between 20 and 69 years old in USA [23].

As expected, men had significantly higher mean excretion of potassium compared to women (*p* = 0.003). This result is similar to findings from Italy and West Africa respectively [24, 25]. Studies from China, Japan, and Iran showed mean urinary potassium excretion among men ranging between 40–50 mmol/day and 40–42 mmol/day among women [17, 26, 27]. This could be attributed by the fact that men generally require higher energy intake compared to women due to their larger body size. Consumption of higher caloric food will subsequently increase potassium intake as well [25, 28].

In this study, urinary potassium excretion was significantly different among the main ethnic groups in Malaysia with respondents from the Chinese ethnic group recorded the highest excretion level. There are no prior data available on potassium intake among the adults in Malaysia to compare with the findings from this study. However, data from the Malaysian Adult Nutrition Survey (MANS) 2014 demonstrated that respondents of Chinese ethnicity had the highest frequency of fruits intake and second highest in frequency of vegetables intake among the ethnic groups in the country [29]. This could explain the reason of higher urinary potassium excretion demonstrated by the Malaysian Chinese in our study since vegetables and fruits are the two main contributors of potassium in the diet [30].

With regard to its relationship to blood pressure level, urinary potassium excretion was not a statistically significant predictor in our study. Studies conducted in China and Netherlands showed a similar finding [18, 19]. However, findings from other studies have suggested urinary potassium excretion does affect blood pressure [9, 10, 17, 20]. It is possible that potassium’s effect in reducing blood pressure occurs only when there is a high potassium intake [10, 18]. In this case, the relationship may not be captured in the MyCoSS sample because 99% of the respondents had their mean urinary potassium excretion lower than the recommended daily intake by WHO (90 mmol) [11]. Despite the absence of statistical significance, our regression output does show a negative association of systolic (β = − 0.15 mmHg) and diastolic blood pressure (β = − 0.12 mmHg) per mmol of potassium excretion.

MyCoSS being the first nationwide study to quantify sodium and potassium excretions using 24-h urine sample in the country is the main strength of this study. Findings from this study will serve as the baseline data on urinary potassium excretion among adults in Malaysia. Moreover, this study applied a reliable method in estimating intake of potassium among the population via 24-h urinary potassium excretion [14]. Few limitations were observed in this study. Firstly, potassium excretion was measured from a single 24-h urine. Data generated from a single 24-h urine analysis is insufficient to describe habitual intake of an individual but it can be a good indicator for average intake of a group of individuals [31]. This approach is acceptable because it is impractical to collect a few completed 24-h urine samples from all respondents in a nationwide study. Secondly, dietary intake of the respondents was not assessed accordingly to study the dietary pattern which causes a very low consumption of potassium-rich food. Data from MANS 2014 showed mean serving size of fruits among the adults was 1.40 servings/day compared to 2.00 servings/day as recommended in Malaysian Dietary Guideline (MDG) 2010. For vegetables, the mean serving size was 1.51 servings/ day compared to 3.00 servings/ day as recommended in MDG 2010 [12]. This shows Malaysians indeed were consuming very low amount of potassium-rich food. Lastly, intake of anti-hypertensive drugs was not included as one of the covariates to be controlled for in multivariable linear analysis to determine the relationship between blood pressure and urinary potassium excretion.

## Conclusions

Findings from this study showed low urinary potassium excretion among respondents which indicates low dietary potassium intake among the adults in Malaysia. Malay adults and females have recorded lower potassium intake in their respective group of analysis. Although potassium intake was not significantly associated with blood pressure in this study, the results from the regression analysis supports potassium’s protective role in blood pressure control. Therefore, the general population in the country need to be educated and encouraged to consume food rich in potassium, particularly fresh fruits and vegetables in their daily diet to maximize the benefits of potassium intake for health. Relevant government agencies should consider providing subsidies in the forms of price control or income assistance to ease the burden of the middle- and low-income groups in purchasing fruits and vegetables.

## Data Availability

The datasets used and/or analyzed during the current study are available from the corresponding author on reasonable requests.

## Abbreviations

MyCoSS: Malaysian Community Salt Survey
RNI: Recommended Nutrient Intake
WHO: World Health Organization
NHMS: National Health and Morbidity Survey
IOM: Institute of Medicine
NHANES: National Health and Nutrition Examination Survey
PURE: Prospective Urban and Rural Epidemiological Study
BMI: Body mass index
SD: Standard Deviation
SPSS: Statistical Package for the Social Sciences
CI: Confidence interval
MDG: Malaysian Dietary Guideline
